# Dual Binding of an Antibody and a Small Molecule Increases the Stability of TERRA G-Quadruplex**

**DOI:** 10.1002/ange.201408113

**Published:** 2014-11-24

**Authors:** Philip M Yangyuoru, Marco Di Antonio, Chiran Ghimire, Giulia Biffi, Shankar Balasubramanian, Hanbin Mao

**Affiliations:** Department of Chemistry and Biochemistry, Kent State University Kent, OH 44242 (USA) E-mail: hmao@kent.edu; Department of Chemistry, University of Cambridge Lensfield Road, CB2 1EW (UK) E-mail: sb10031@cam.ac.uk; Cancer Research UK, Cambridge Research Institute Li Ka Shing Centre, Robinson Way, Cambridge CB2 0RE (UK); School of Clinical Medicine, University of Cambridge, Addenbrooke's Hospital Hills Road, Cambridge CB2 0SP (UK)

**Keywords:** Einzelmolekülstudien, G-Quadruplexe, Ligandeneffekte, Optische Fallen, RNA-Strukturen

## Abstract

In investigating the binding interactions between the human telomeric RNA (TERRA) G-quadruplex (GQ) and its ligands, it was found that the small molecule carboxypyridostatin (cPDS) and the GQ-selective antibody BG4 simultaneously bind the TERRA GQ. We previously showed that the overall binding affinity of BG4 for RNA GQs is not significantly affected in the presence of cPDS. However, single-molecule mechanical unfolding experiments revealed a population (48 %) with substantially increased mechanical and thermodynamic stability. Force-jump kinetic investigations suggested competitive binding of cPDS and BG4 to the TERRA GQ. Following this, the two bound ligands slowly rearrange, thereby leading to the minor population with increased stability. Given the relevance of G-quadruplexes in the regulation of biological processes, we anticipate that the unprecedented conformational rearrangement observed in the TERRA-GQ–ligand complex may inspire new strategies for the selective stabilization of G-quadruplexes in cells.

Non-canonical nucleic acid structures such as G-quadruplexes have recently attracted significant attention for their potential roles in the regulation of biological processes.[1] G-quadruplexes are formed with a minimum of four guanine (G)-rich repeats in DNA or RNA sequences.[2] They consist of a stack of planar guanine tetramers called G-quartets that are stabilized by Hoogsteen hydrogen bonds and monovalent cation coordination.[3] G-quadruplex-forming sequences are prevalent in the human genome[4–6] and are particularly enriched at telomeres and in the promoter regions of genes.[7, 8] The formation of stable G-quadruplex structures in the telomeric 3′ overhang has been shown to have an inhibitory effect on telomerase, an enzyme up-regulated in a majority of cancer cells.[9] Therefore, the design of small-molecule ligands that can selectively bind and stabilize DNA G-quadruplexes (GQs) in cells has been intensively investigated as a potential strategy for cancer therapy.[10] It has been shown that mammalian telomeres can be transcribed into telomeric repeat-containing RNA (TERRA),[11] which can also form G-quadruplexes in vivo.[12] More generally, RNA G-quadruplexes have been shown to regulate biological processes such as translation.[13, 14] This provides a new route to control these biological processes by using molecules that selectively bind to the RNA GQs. We recently demonstrated that RNA G-quadruplexes can form in the cytoplasm of cells and that they can be stabilized and visualized by the selective RNA GQ ligand carboxypyridostatin (cPDS)[15] and the antibody BG4.[16] These observations have led to the possibility of a multifaceted regulatory approach, for example, through antibody–drug conjugates (ADCs).[17] It is still unclear whether BG4 and cPDS can cooperatively bind and stabilize an RNA GQ structure in a cellular context. A ternary complex in which an antibody and a small molecule can cooperatively stabilize a GQ would offer a novel approach to target and stabilize these structures and support the observed increase in BG4 staining upon treatment with G-quadruplex ligands. Furthermore, it would be of great significance to see whether the conformational rearrangement widely observed in proteins[18–20] and other nucleic acid structures[21, 22] are also observed upon binding of ligands to G-quadruplexes.

In this report, we investigated the dual binding of cPDS and the BG4 antibody to the TERRA G-quadruplex. Using a mechanical unfolding approach with laser tweezers, we found that a minor TERRA G-quadruplex population (48 %) has increased mechanical and thermodynamic stability when bound to both ligands. With force-jump kinetic investigations, we revealed that the two ligands compete for the binding initially, followed by a slow rearrangement that leads to the formation of the ternary complex. This behavior suggests a conformational transition during binding, which leads to increased stability of the bound TERRA GQ. We anticipate that this new binding strategy may inspire the development of ligands with more effective binding to specific G-quadruplex structures.

To carry out single-molecule mechanical unfolding experiments, the GQ-forming sequence 5′-UUA(GGG UUA)_4_-3′ (TERRA-G4) was sandwiched between two double-stranded DNA/RNA hybrid spacers, which were separately attached to two optically trapped polystyrene beads in a laser tweezers instrument. The entire nucleic acid construct was mechanically stretched and relaxed (Figure 1 A) in a 10 mm Tris buffer (pH 7.4) that contains 100 mm KCl at 23 °C in a microfluidic chamber. Unfolding events, indicated by a sudden change in contour length (Δ*L*) during mechanical stretching, were recorded in force–extension (*F*–*X*) curves (Figure 1 B). Using probability density distribution of Δ*L* and bootstrap statistical analyses (PoDNano,[23] see the Supporting Information), the Δ*L* histograms were deconvoluted into two major populations with Gaussian centers at 9.4±0.2 and 5.7±0.3 nm (Figure 1 B inset). Consistent with earlier reports,[24] these Δ*L* populations represent a GQ structure (9.4 nm) and partially folded species (5.7 nm). The latter may assume a G-Triplex[25, 26] conformation. Similar Δ*L* populations were observed when the TERRA-G4 was incubated with the small-molecule ligand cPDS or the antibody BG4 (Figure 2, bottom panels), which implies that these binding partners do not significantly disrupt the formation of GQ or the G-Triplex intermediate. In fact, there is a slight increase in the partially folded population for the antibody and cPDS mixture, thus suggesting that various intermediates are present as a result of multiple binding pathways.

**Figure 1:**
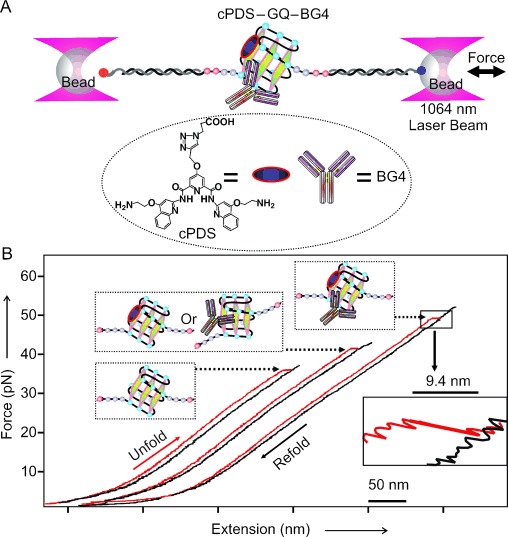
Single-molecule mechanical unfolding and refolding experiments. A) Laser tweezers set-up, in which a single-stranded RNA containing a TERRA-4G sequence is sandwiched by two DNA–RNA hybrid handles attached to two optically trapped beads. Inset shows the structure of carboxypyridostatin (cPDS). B) Typical *F–X* curves show different rupture forces for the G-quadruplexes bound to the small molecule cPDS, the antibody, or both. Inset shows a blown-up region of the rupture event.

**Figure 2:**
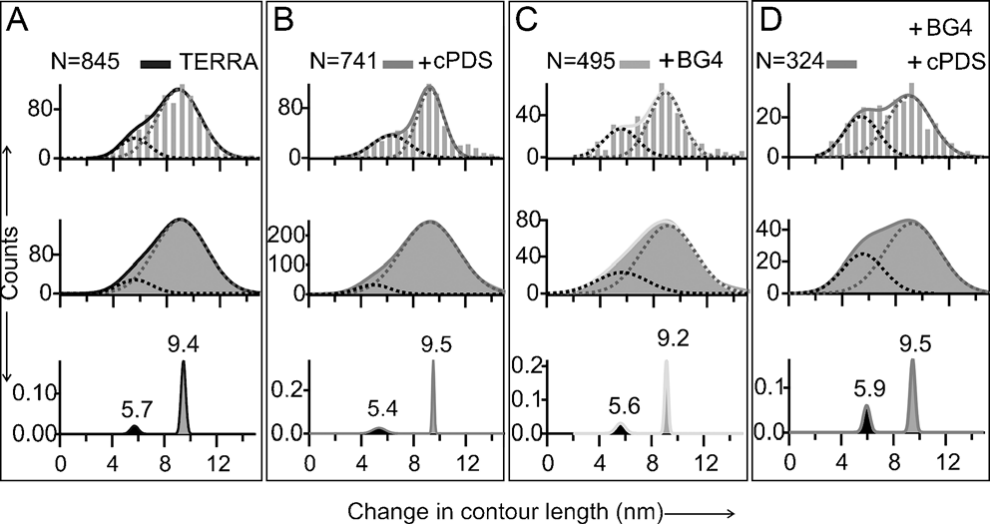
Changes in contour length (Δ*L*) of the structures in the TERRA construct. Top panels: Δ*L* histogram, middle: Kernel density distribution, bottom: PoDNano of the kernel density distribution. TERRA without ligands (A) or with 5 μm cPDS (B), 50 nm BG4 (C), or 50 nm BG4+5 μm cPDS (D). Solid curves depict Gaussian fitting.

Next, we investigated the mechanical stability of the TERRA GQ bound to the antibody or cPDS. Previous studies have shown that DNA GQs bound to ligands have increased mechanical stability compared to free GQs.[27] Depending on the time of measurement or the concentration of the ligands, however, the fraction of bound GQ varies. To ascertain the mechanical stability of bound TERRA species more accurately, we analyzed the rupture forces of folded structures when different species reached equilibrium after approximately 45 seconds of refolding, which was carried out at 0 pN after mechanical unfolding of the structures formed in the TERRA-G4 fragment (Figure 3, see the Supporting Information for experimental details).

**Figure 3:**
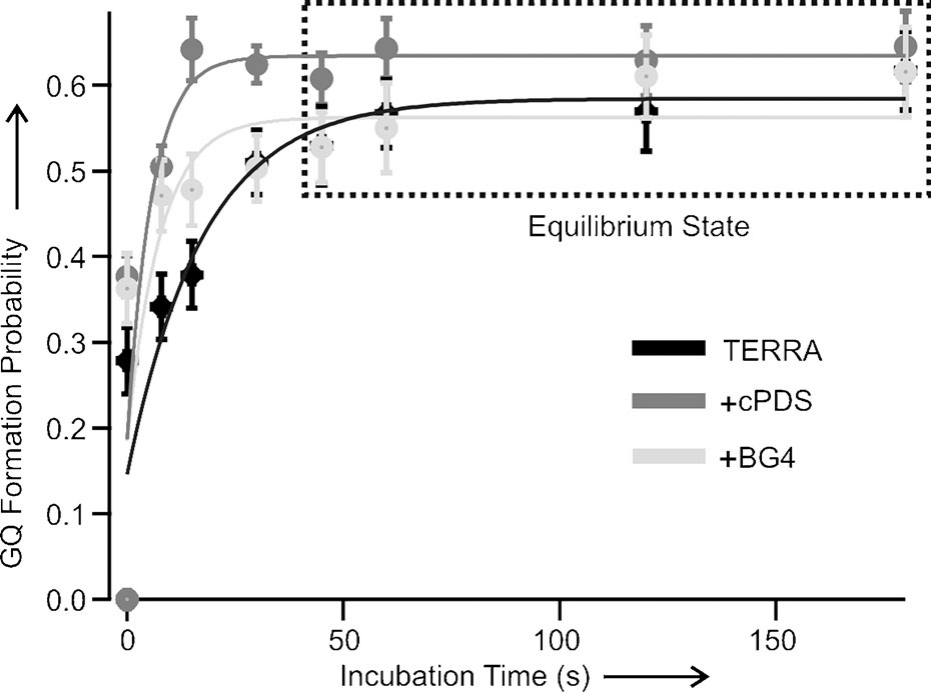
Probability of G-quadruplex (GQ) formation against incubation time for TERRA without ligand (black), with cPDS (dark gray), or with BG4 (light gray). The highlighted plateau indicates the equilibrated folding state. Solid curves represent fitting from a two-state model (see the Supporting Information).

After deconvoluting the GQ and the partially folded species,[25] the mechanical stability of each species was analyzed in separate rupture-force histograms (Figure 4 for GQ and Figure S1 in the Supporting Information for partially folded species). The rupture-force histogram for the TERRA GQ without ligands shows more than one population (Figure 4 A), representing multiple conformations in the same TERRA sequence as reported previously.[24, 28] When the TERRA construct was incubated with 5 μm cPDS, we observed an increase in the rupture forces (from 23/36 pN to 25/40 pN), thus suggesting that the binding of cPDS increases the mechanical stability of the TERRA GQ (Figure 4 B). A similar increment in mechanical stability was observed for TERRA-G4 with 50 nm BG4 (Figure 4 C). Interestingly, with a mixture of 5 μm cPDS and 50 nm BG4, we observed two populations with rupture forces centered at 30 and 50 pN (Figure 4 D), respectively. Compared to the populations in the presence of either cPDS or BG4, these two populations showed increased rupture forces. Whereas the 30 pN species can be contributed from the higher force population in either cPDS or BG4 solutions, the 50 pN population (48 %) clearly suggests that the TERRA GQ has two separate binding sites to simultaneously accommodate BG4 and cPDS. Analyses of the change in the free energy of unfolding (Δ*G*_unfold_) confirmed that this species is thermodynamically more stable (17, 17, and 25 kcal mol^−1^ for cPDS, BG4, and cPDS+BG4 bound to TERRA GQs, respectively, see Table S1 and Figure S2 in in the Supporting Information). In fact, the extra Δ*G*_unfold_ gained from the simultaneous binding of the two ligands (ΔΔ*G*_unfold_=9.1 kcal mol^−1^) is more than the sum of those from the binding of cPDS or BG4 alone (1.3+1.4=2.7 kcal mol^−1^). This fact indicates the presence of additional interactions between the two binding sites, probably as a result of conformational rearrangement during binding of the ligands, a characteristic of allosteric effects. It is noteworthy that the rupture-force histograms of the partially folded species remained unaffected after incubation with cPDS, BG4, or both (Figure S1), thus suggesting that the ligand or antibody binds to the TERRA GQ rather than a partially folded TERRA species such as a G-Triplex. Consistent with what was observed in the Δ*L* populations (Figure 2 D), a slight increase in the low-rupture-force population was observed in the presence of both cPDS and BG4 ligands, which again suggests the presence of multiple intermediates as pathways to ligand binding become more complex (Figure 4).

**Figure 4:**
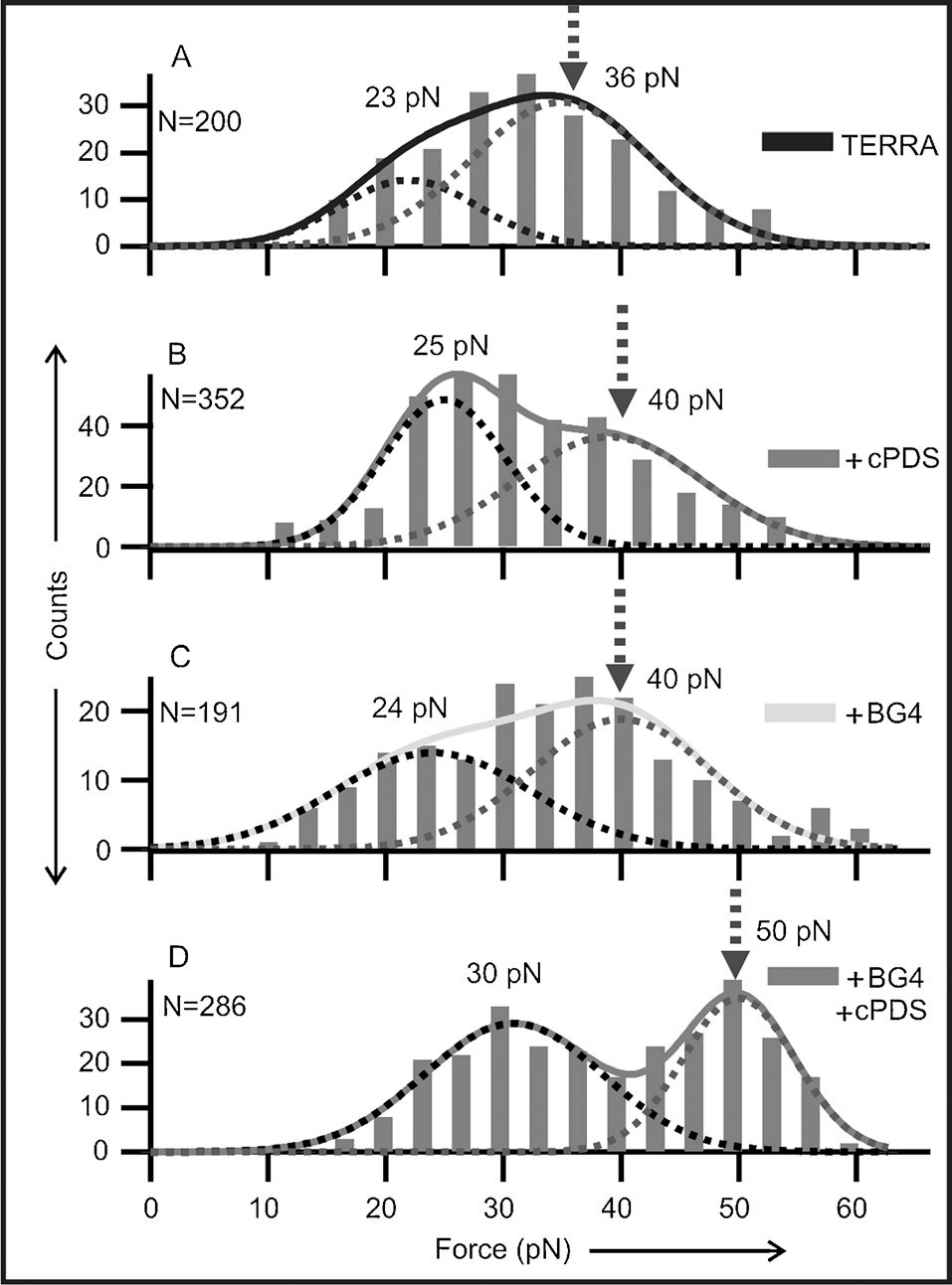
Rupture-force histograms for TERRA G-quadruplexes without ligands (A), with 5 μm cPDS (B), with 50 nm BG4 (C), or with 5 μm cPDS+50 nm BG4 (D). Gaussian fitting (dotted curves) reveal two major populations. The higher force populations are indicated by dotted arrows.

To follow individual species with a better temporal resolution, we performed single-molecule kinetic experiments using the force-pumping and force-probing approach.[27, 29] As soon as we mechanically unfolded TERRA GQs, we relaxed the force to 0 pN within 10 ms to allow the GQs to refold (force-pumping). The folding of the structure during incubation is probed by the next round of the force-ramping procedure (force-probing). As shown in Figures 3 and 5, while cPDS provides the biggest increase in folding rate for the TERRA GQ, the antibody BG4 has the least effect. When both cPDS and BG4 are present, an intermediate increase in the folding rate was observed. If the binding of cPDS and BG4 were not competitive, the formation of the GQ would follow the fastest binding kinetics determined by cPDS alone. Therefore, our results indicate that BG4 competes with cPDS for initial binding.

**Figure 5:**
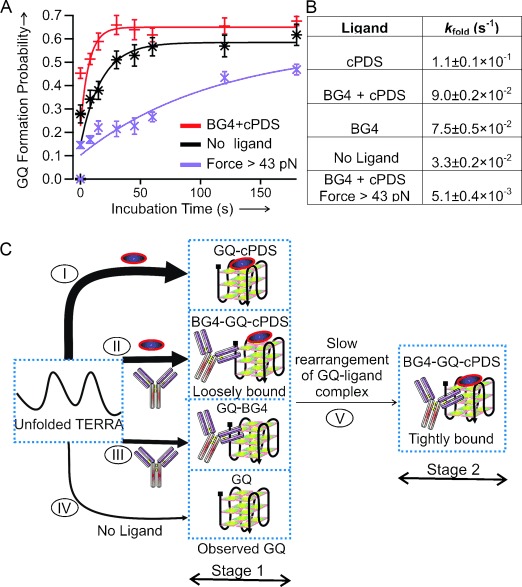
Two-stage binding of cPDS (5 μm) and BG4 (50 nm) to the TERRA G-quadruplex. A) Folding kinetics of the TERRA G-quadruplex in solutions without ligands (black) or with cPDS+BG4 (red). The purple data points depict the folding kinetics of the species with rupture force>43 pN in the presence of both BG4 and cPDS. B) Folding rate constants (*k*_fold_) of TERRA G-quadruplexes under different conditions. C) Schematic of the proposed two-stage binding. The formation rate constant for G-quadruplex is indicated by the thickness of each arrow.

Since the GQ bound to both cPDS and BG4 (rupture force>43 pN) can be clearly differentiated from the singly bound species or free quadruplexes, we deconvoluted this species (rupture force>43 pN) and followed its folding kinetics. As shown in Figure 5 A, B, the folding kinetics of this species is slowest even compared to that of free TERRA GQ. Based on the kinetics observed in the presence of different ligands, we reconstructed the folding pathways of TERRA GQ in a two-stage process in the cPDS and BG4 mixture (Figure 5 C). In the first stage, formation of the GQ is mediated by competitive binding of cPDS and BG4. Since folding of the GQ is fastest in the presence of cPDS, the cPDS-bound GQ (process I in Figure 5 C) represents the predominant species at the end of this stage. In the second stage, the cPDS-bound species started to accept BG4 and slowly rearranged to finalize the binding complex for both cPDS and BG4 (process V in Figure 5 C). As a result, the ternary complex becomes more stable both mechanically and thermodynamically, the latter of which was suggested previously by immunofluorescence[16] and demonstrated in this work by means of the optical tweezers measurements (Table S1) and FRET-melting experiments (Figure S3). Similar synergistic effects from different ligands have been observed in both ensemble[30] and single-molecule experiments.[31]

In summary, we have observed conformational rearrangement during the simultaneous binding of the GQ-selective BG4 antibody and the small molecule cPDS to the TERRA GQ. We anticipate that the increased mechanical and thermodynamic stability resulting from the conformational rearrangement could provide new leads for the design of more effective GQ-binding ligands.

## Supporting Information

As a service to our authors and readers, this journal provides supporting information supplied by the authors. Such materials are peer reviewed and may be re-organized for online delivery, but are not copy-edited or typeset. Technical support issues arising from supporting information (other than missing files) should be addressed to the authors.

miscellaneous_information
